# Caulerpa lentillifera improves ethanol-induced liver injury and modulates the gut microbiota in rats

**DOI:** 10.1016/j.crfs.2023.100546

**Published:** 2023-07-08

**Authors:** Kuan-Yu Lin, Hsin-Yi Yang, Suh-Ching Yang, Ya-Ling Chen, Y. Watanabe, Jiun-Rong Chen

**Affiliations:** a School of Nutrition and Health Sciences, Taipei Medical University, Taipei, Taiwan; b Department of Nutritional Science, Fu Jen Catholic University, New Taipei City, Taiwan; c General Health Medical Center, Yokohama University of Pharmacy, Yokohama, Japan; d Nutrition Research Center, Taipei Medical University Hospital, Taipei, Taiwan

**Keywords:** Alcoholic liver disease, Sea grape, Inflammation, Microbiota, TLR4

## Abstract

*Caulerpa lentillifera* (CL), also called sea grape, is a type of edible green alga which was reported to have antioxidative and immunomodulatory potential. This study aimed to investigate the hepatoprotective effects of CL in a rat model of chronic ethanol exposure. Wistar rats were assigned to four groups and supplied with an isocaloric control liquid diet (group C), an ethanol liquid diet (group E), a control liquid diet supplemented with 5% CL (group CC), or an ethanol liquid diet supplemented with 5% CL (group EC) for a 12-week experimental period. Ethanol feeding induced steatosis, inflammation, and changes in the gut microbiota by the end of the study, whereas CL supplementation significantly improved liver injuries and decreased circulatory endotoxin levels. Moreover, we also found that CL reversed ethanol-induced elevation of hepatic toll-like receptor 4 (TLR4), MyD88 protein expression, the phosphorylated-nuclear factor (NF)-κB-to–NF–κB ratio, and proinflammatory cytokine concentrations. Additionally, CL also increased the abundance of *Akkermansia* and tight junction proteins and diminished the *Firmicutes*-to-*Bacteroidetes* ratio. Dietary CL inhibited the progression of alcoholic liver disease, and some of the possible mechanisms may be strengthening the intestinal barrier function, alleviating dysbiosis, and modulating the TLR4 pathway.

## Highlights

• *Caulerpa lentillifera* ameliorated alcoholic liver inflammation.
• *Caulerpa lentillifera* suppressed the hepatic TLR4-Myd88 signaling pathway.
• *Caulerpa lentillifera* increased the tight junction protein.
• *Caulerpa lentillifera* the abundance of genus *Akkermansia.*

## Graphical abstract

**Figure:**
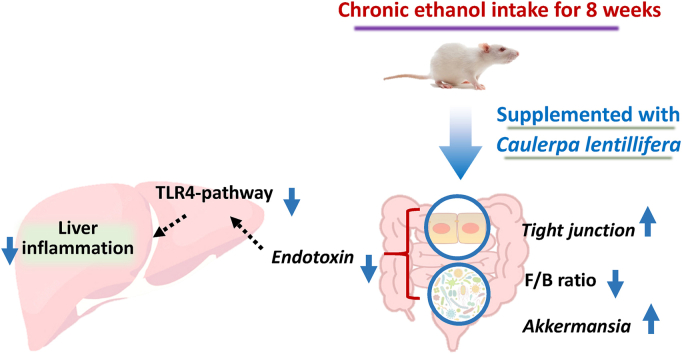

## Introduction

Ethanol consumption is a predominant cause of chronic liver diseases worldwide (Miller et al., 2011). Excessive ethanol use is a critical factor that results in diverse ethanol-associated diseases (WHO, 2018), and ethanol abuse has become more serious because of attributable risk factors that exist among adults aged less than 60 years (Lo et al., 2017). Alcoholic liver disease (ALD) comprises steatosis, hepatitis, fibrosis, cirrhosis, and hepatocellular carcinoma. Steatosis and hepatitis are reversible after one stops drinking, but with continued drinking, the disease could progress towards fibrosis and cirrhosis or even a hepatic carcinoma. Cederbaum (2012) suggested that chronic ethanol intake activates hepatic cytochrome P450 2E1 (CYP2E1) expression, and CYP2E1 also plays a critical role in reactive oxygen species (ROS) generation during ethanol metabolism. With chronic alcohol consumption, hepatic lipids accumulate, oxidative stress increases, and inflammation cascades are upregulated (Lu and Cederbaum, 2008; Wang et al., 2013). In addition, recent reports also demonstrated that excess ethanol intake may result in changes in tight junction protein expressions and dysbiosis, which induce bacterial translocation that activates hepatic inflammation responses in vivo through the gut-liver axis (Bermingham et al., 2017). In addition to lifestyle modifications and medical treatments, many food-derived bioactive compounds have drawn attention for their potential against ALD progression (Osna et al., 2017).

*Caulerpa lentillifera* (CL), also called sea grape, is an edible green seaweed, classified as a species of bryopsidale green algae from tropical and subtropical coastal water area such as Australia, Japan, Thailand, or Indonesia. It is also widely cultivated in eastern Taiwan and is an alternative food that can also be used therapeutically (Nguyen et al., 2011; Leandro et al., 2020). Owing to its nutritional value, potential pharmacological benefits, and sustainability, CL is regarded as a functional food that can potentially be a beneficial and alternative therapy for human health (Nofiani et al., 2018; Paul et al., 2014). An increasing number of studies have shown that CL exhibits lipid-lowering (Sharma and Rhyu, 2014), immunomodulatory (Maeda et al., 2012), and antioxidative properties (Nguyen et al., 2011). However, the impacts of dietary CL on excessive ethanol-induced steatosis and hepatic injuries remain unclarified. Therefore, the aim of the study was to investigate the effects of CL supplementation on ALD in a rat model that mimics chronic alcohol intake in humans and clarify possible mechanisms.

## Materials & methods

### Experimental design

Six-week-old male Wistar rats were purchased from BioLASCO Taiwan (Taipei, Taiwan). All animal protocols were reviewed and approved by the Institutional Animal Care and Use Committee (IACUC) of Taipei Medical University (approval no.: LAC-2017-0522) and were in accordance with the eighth edition of the Guide for the Care and Use of Laboratory Animals. Animals were housed in groups of two rats per cage at the Laboratory Animal Center (Taipei Medical University) at a content temperature of 22 ± 2 °C and humidity of 55% ± 5% with a 12-h light/dark cycle. After a 2-week acclimation period, rats were randomly divided into four experimental groups (n = 8) and fed with different liquid diet as shown in Supplementary Table 1: a control group (C, isocaloric control liquid diet, 39.4% of calories from carbohydrates), an ethanol group (E, 5.4% of calories from carbohydrates and 34% of calories from ethanol), and a control group and ethanol group supplemented with 8.4 (g/L) CL dried powder (CC and EC groups, respectively). Freeze-dried CL powder was obtained from East Green Bio Corporation (Hualien, Taiwan), which is a commercial product for human consumption. It contained (in % dry weight) 16% moisture, 44% carbohydrate, 14% lipid, 7% protein and 18% total dietary fiber, and the dosage was based on a previously published effective amount (Matanjun et al., 2010). For pair feeding, the rats in each group were fed the same amount of food. We recorded food intake every day and the amount of food provided to each group of rats was matched to that consumed by the E group. After a 12-week experimental period, 12-h-starved rats were anesthetized with a Zoletil® (50 mg/kg BW, virbac, Carros, France)-Rompun® (20 mg/kg BW, Bayer, Leverkusen, Germany) mixture (1:1, 1 mL/kg BW, *ip*). Blood, liver, the small intestines, and a fresh stool were collected and stored at −80 °C until being analyzed.

### Blood collection and analysis

Blood samples were collected from the abdominal aorta, separated into serum, and stored at −80 °C. Serum aspartate aminotransferase (AST), alanine aminotransferase (ALT), total cholesterol (TC), and triglycerides (TGs) were determined by utilizing a Roche Modular P800 (Roche Diagnostics, Indianapolis, IN) (Bulut et al., 2017). The activity of γ-glutamyl transpeptidase (GGT) and the endotoxin level were analyzed with commercial assay kits (Randox GT2750, Antrim, UK) and Limulus Amebocyte Lysate test kit (GenScript, L00350, 133 Piscataway, NJ), respectively (Szasz 1969; Lindsay et al., 1989). According to a thiobarbituric acid (TBA)-reactive substance (TBARS) colorimetric method, serum malondialdehyde (MDA) was measured. Briefly, serum samples were reacted with 2-TBA to generate colored end products which were detected spectrophotometrically at 532 nm (Ohkawa et al., 1979).

### Liver collection and analysis

Liver tissues were cut into small pieces and fixed in 10% neutral buffered formalin (NBF). Liver specimens were shaped, sectioned, stained with hematoxylin and eosin (H&E), and examined under a microscope by a veterinarian blinded to the treatment of each group (Kleiner et al., 2005). Hepatic TC and TG levels were extracted with a chloroform: methanol (2:1) solution (Lees and Sloanestanley, 1957) and measured with a commercial assay kit (Randox TR210 & Fortress BXC0271, Antrim, UK). Liver samples were weighed, cut into small pieces, and homogenized in an ice-cold solution of 50 mM Tris-HCl, 150 mM NaCl, 1% NP-40, and 0.1% sodium dodecylsulfate (SDS) with protease inhibitors. After centrifugation at < 4 °C and 3000×*g* for 15 min, we used the supernatant to analyze hepatic MDA levelsand proinflammatory cytokine concentrations (tumor necrosis factor (TNF)-α and interleukin (IL)-1β) (Yoshimura et al., 1997). MDA levels were assessed by a TBARS colorimetric method at 532 nm (Tsikas 2017). TNF-α and IL-1β concentrations were determined using enzyme-linked immunosorbent assay (ELISA) kits (DuoSet® ELISA Development System, DY501 & 510, R&D Systems, Abingdon, UK). Protein concentrations of the supernatants were evaluated with Bio-Rad protein assay dye (BioRad Laboratories, 500–0002, CA). To determine the protein expression of ethanol-inducible cytochrome P450 2E1 (CYP2E1), liver samples were homogenized in a 0.25 M sucrose, 10 mM Tris-HCl, 0.25 mM phenyl-methyl-sulfonyl fluoride mixture and centrifuged at 105,000×*g* for 1 h to obtain microsome pellets. After being resuspended in buffer, microsome samples containing 30 g protein were fractionated by SDS-polyacrylamide gel electrophoresis (PAGE) and transferred to a polyvinylidene difluoride (PVDF) membrane utilizing a transfer apparatus according to the manufacturer's protocols (BioRad) (Fernandez et al., 1998). Blots were incubated with primary antibodies: CYP2E1 (anti-cytochrome P450 2E1 antibody [ab28146], Abcam, Cambridge, UK), followed by corresponding species of secondary antibodies. Bands were scanned on the BioSpectrum AC Image system, UVP Visionwork LS Software and analyzed by Image-Proplus software (Chen et al., 2013). GAPDH (Proteinech, 10494-1-AP, Rosemont, IL) was assessed as the loading control. Expressions of sterol response element-binding protein 1 (SREBP1; SREBP-1 antibody [GTX79299], GeneTex, Irvine, CA), peroxisome proliferator-activated receptor (PPAR)-α (anti-PPAR alpha antibody [ab8934], Abcam), Toll-like receptor 4 (TLR4; monoclonal antibody (mAb) to TLR4 [IMG-5031A], Imgenex, San Diego, CA), MyD88 (MyD88 [D80F5] rabbit mAb, Cell Signaling Technology, Danvers, MA), toll/IL-1 receptor domain-containing adaptor protein-inducing interferon β (TRIF) (TRIF/TICAM1 antibody [NB120-13810], Imgenex) protein levels, and the p–NF–κB-to–NF–κB ratio (phosphorylated–NF–κB p65 (Ser536) (93H1) rabbit mAb [3033], Cell Signaling Technology; NF-κB p65 antibody [622,601], Biolegend, San Diego, CA) were determined by Western blotting (Wang et al., 2013). GAPDH and proliferating cell nuclear antigen (PCNA) were detected as the loading controls.

### Small intestine collection and analysis

To evaluate the gut barrier integrity, small intestine samples were weighed, cut into small pieces, and homogenized in an ice-cold solution of 50 mM Tris-HCl, 150 mM NaCl, 1% NP-40, and 0.1% SDS with protease inhibitors (Costantini et al., 2010). Occludin (occludin polyclonal antibody [QC216103], Thermo Fisher, Waltham, MA) and zonula occludens protein 1 (ZO-1; ZO-1 polyclonal antibody [217731AP], Proteintech, Rosemont, IL) were assessed, and β-actin was chosen as the internal control.

### Stool collection and analysis

All procedures and analyses of sequencing were entrusted to BIOTOOLS (New Taipei City, Taiwan). In brief, bacterial DNA was extracted using a QIAamp DNA Stool Mini Kit (Qiagen, ST) according to Godon's method (Godon et al., 1997). After extraction, V3–V4 regions of 16S ribosomal (r)DNA were amplified with Illumina® HiSeq Sequencing to obtain raw data. Sequences were generated as clusters using UCHIME (Quast et al., 2012) and filtered from the effective tags (>97% sequence identity) by utilizing USEARCH software (vers. 7.0.1090). Organization taxonomic unit (OTU) abundance information was selected to achieve minimum sequences per sample to avoid sampling depth bias. To evaluate the cecal microbiotic richness, evenness, and diversity, alpha diversity was calculated. a beta diversity examination was used to examine variations in the species complexity of samples. A principal component analysis (PCA) was applied for compression and classification of data into different groups (Koleff et al., 2003). To determine differences in each group, relative abundances of microbial species at the phylum and genus levels are displayed. The Firmicutes-to-Bacteroidetes (F/B) ratio is also presented in box plots. On the other hand, sequences per samples were annotated by taxonomic classification according to the RDP classifier (vers. 2.11) (Hong et al., 2016). LEfSe (Linear discriminant analysis Effect Size) was performed to determine significant taxa or biomarkers among the four groups using relative abundances with a non-parametric factorial Kruskal-Wallis rank-sum test. The length of the bar constitutes a log10 altered linear discriminant analysis (LDA) score (default: 4). A cladogram and LDA scores of bacterial communities of each group were drawn using QIIME.

### Statistical analysis

Values are presented as the mean ± standard error of the mean (SEM). Statistical analysis was carried out using SAS software version 9.4 for Windows (SAS, Cary, NC). Students' *t*-test was used to compare the difference between initial and final values of AST, ALT, and GGT activities in the same group. On the other hand, a one-way analysis of variance (ANOVA) followed by Duncan's multiple-range test was used to identify the differences all groups. A two-way ANOVA was used to confirm the interaction between ethanol and CL. Correlation coefficients between relative protein expressions and intestinal bacterial species were evaluated using a Pearson correlation analysis. A P value of <0.05 was considered significantly significant.

## Results

### Body weight and food intake

No significant difference in the initial BW at the baseline was found among groups (*P* > 0.05). After the 12-week experimental period, we found the final BWs in the two ethanol-consuming groups were significantly lower than that of the C group (*P* < 0.05), while no difference between the E and EC groups was found. In addition, we found no difference in daily energy intake among the four groups (*P* > 0.05). Furthermore, we also found no difference in daily ethanol intake between the E and EC groups and no difference in the CL intake between the CC and EC group (Supplementary Table 2).

### Ethanol-induced liver injury

**Fig. 1:**
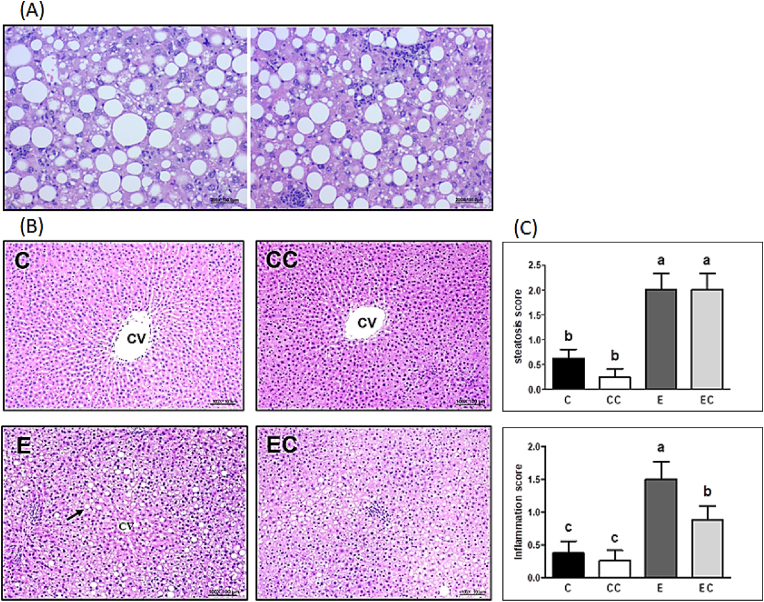
Histopathological analysis (H&E). (A) ethanol-induced steatosis, inflammation and hepatocellular ballooning of the E group (200X) (B) liver section of rats at the end of the study (100X) (C) steatosis and inflammation score. Arrow indicates the accumulation of lipid droplet. Values are presented as the mean ± SEM based on 8 rats in each group. Significant difference (p < 0.05) is identified by different letters. C, control group; CC, control + CL group; E, ethanol group; EC, ethanol + CL group; CV, central vein.

The serum AST and ALT activities did not differ among the four groups at the baseline (*P* > 0.05, Table 1). After 12 weeks of ethanol feeding, serum AST, ALT and GGT activities were significantly increased in the E group, while EC group only represented the significantly higher serum AST and ALT activities. When compared with the C group, the AST, ALT and GGT activities were significantly elevated in the E group. However, the AST and GGT activities were significantly reduced in the EC group when compared with the E group (*P* < 0.05). The liver histological evaluation was shown in Fig. 1. The E group showed obvious diffuse steatosis, focal cellular ballooning and lobular inflammation steatosis in liver sections while the C and CC group only showed minimal sign of steatosis or inflammation. The steatosis in group E begins in the centrilobular Zone 3 and progresses towards the periportal Zone 1. It began with small droplets of fat in the cytoplasm, which later enlarged to large fat droplets and pushed the nucleus to the periphery. Likewise, the E group exhibited a higher steatosis score and inflammation score compared to the C group. The EC group supplemented with CL exhibited a decline in the inflammation score compared to the E group. Above all, the results demonstrated that CL supplementation may improve ethanol-induced liver injury (Fig. 1).

**Table 1:** Effects of *Caulerpa lentillifera* (CL) on ethanol-induced liver injury in rats subjected to chronic ethanol consumption.

	C	CC	E	EC
AST (IU/L)				
Initial	98.5 ± 5.5	96.4 ± 3.2	108.3 ± 11.2	99.9 ± 7.1
Final	129.5 ± 7.6 ^c^	110.3 ± 4.5 ^c^	238.1 ± 17.3 *^a^	198.9 ± 29.0 *^b^
ALT (IU/L)				
Initial	28.0 ± 0.9	26.1 ± 1.4	28.4 ± 2.0	27.0 ± 2.0
Final	31.9 ± 3.3 ^b^	30.8 ± 2.7 ^b^	95.3 ± 11.2 *^a^	103.6 ± 18.5 *^a^
GGT (U/L)				
Initial	0.7 ± 0.1	0.8 ± 0.2	0.7 ± 0.1	0.7 ± 0.1
Final	0.8 ± 0.2 ^b^	1.1 ± 0.1 ^b^	2.0 ± 0.1 *^a^	1.0 ± 0.1 ^b^

Values are presented as the mean ± SEM based on 8 rats in each group. Asterisk (*) indicates the differences between initial and final values in the same groups using Students' *t*-test. Significant difference (p < 0.05) is identified by different letter. C, control group; CC, control + CL group; E, ethanol group; EC, ethanol + CL group; AST, aspartate transaminase; ALT, alanine transaminase; GGT, gamma-glutamyl transpeptidase.

### Ethanol mediated abnormal lipid metabolism

**Fig. 2:**
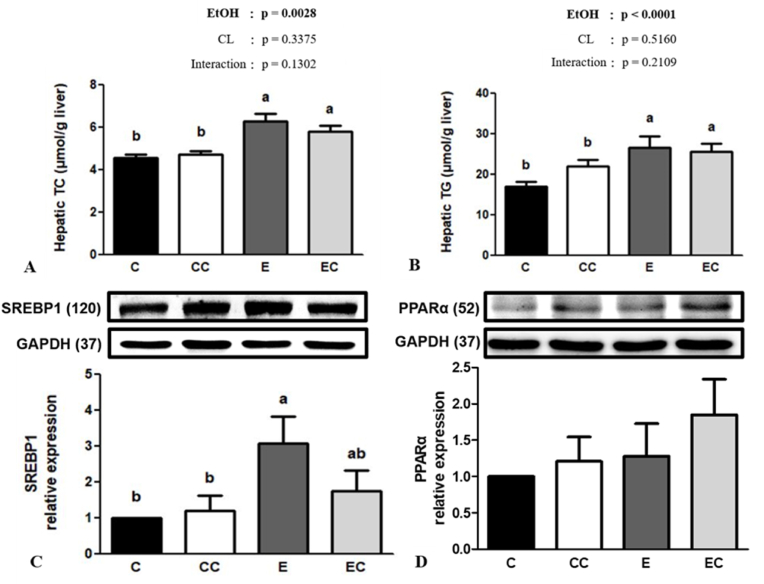
Effect of *C. lentillifera* on liver homeostasis (A) total cholesterol (TC) and (B) triglyceride (TG) levels, and relative hepatic (C) sterol regulatory element-binding protein 1 (SREBP1c) and (D) peroxisome proliferator-activated receptor alpha (PPARα) protein expressions. Values are presented as the mean ± SEM based on 8 rats in each group. Significant difference (p < 0.05) is identified by different letters. C, control group; CC, control + CL group; E, ethanol group; EC, ethanol + CL group.

In the analysis of hepatic lipids, we found no significant ethanol and CL interaction in hepatic lipid levels (*P* > 0.05). Nevertheless, we found ethanol consumption significantly elevated hepatic TC and TG concentrations while CL supplementation showed no significant effects in both parameters (Fig. 2). Additionally, CL supplementation exhibited no difference in the relative protein expressions (SREBP1 and PPARα) associated with liver lipid metabolism (*P* > 0.05).

### Ethanol-induced oxidative stress

MDA, a serum lipid peroxidation product, displayed no difference among the four groups. There were no significant impacts of ethanol × CL interaction on serum MDA (*P* = 0.1450) and hepatic MDA (*P* = 0.5426) levels. We also found no significant effects of CL supplementation in both serum and hepatic MDA concentrations during ethanol consumption (*P* > 0.05). In addition, the EC group tended to have lower hepatic CYP2E1 protein expression than the E group, but no significant difference was found (Supplementary Fig. 1).

### Ethanol-induced inflammation

**Fig. 3:**
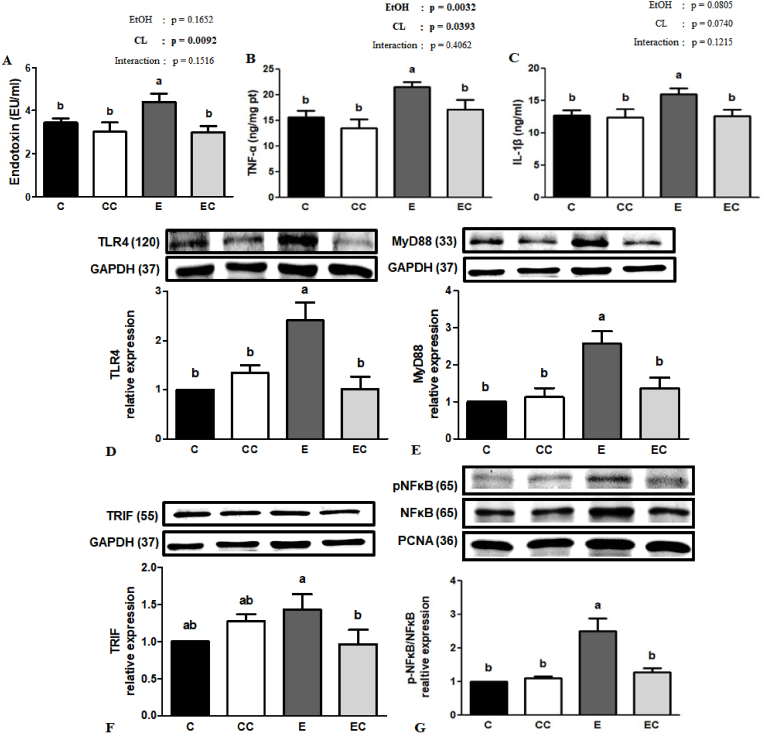
Effect of *C. lentillifera* on serum (A) endotoxin and hepatic (B) TNF-α and (C) IL-1β levels, and (D) relative Toll-like receptor 4 (TLR4), (E) myeloid differentiation primary response gene 88 (MyD88), (F) TIR-domain-containing adapter-inducing interferon-β (TRIF), and (G) phosphorylated (p)-nuclear factor kappa light chain enhancer of activated B cells (NF-κB)/NF-κB protein expressions analyzed by western blotting. Values are presented as the mean ± SEM based on 8 rats in each group. Significant difference (p < 0.05) is identified by different letters. C, control group; CC, control + CL group; E, ethanol group; EC, ethanol + CL group.

Many studies have reported that blood endotoxin levels are increased under ethanol administration, which aggravates liver injury (Osna et al., 2017). We determined serum endotoxin levels at the end of the study and found that group E showed significant increase of circulatory endotoxin concentrations compared to the C group (*P* < 0.05). Although no significant ethanol and CL interaction was found, group EC had significant lower endotoxin level than the E group (*P* < 0.05). Additionally, we found no significant ethanol and CL interaction in hepatic proinflammatory cytokines (*P* > 0.05). Ethanol consumption significantly elevated hepatic TNF-α and IL-1β level and group EC showed significant lower TNF-α and IL-1β than group E. To clarify the possible mechanisms of CL treatment against ethanol-induced liver inflammation, relative protein expressions of the TLR4 signaling pathway were determined. As shown in Fig. 3, hepatic TLR4 and MyD88 protein expressions and the p–NF–κB-to–NF–κB ratio were significantly increased compared to the C group, while CL supplementation downregulated ethanol-induced elevation of TLR4, MyD88, and TRIF protein expressions and the p–NF–κB-to–NF–κB ratio.

### Ethanol-induced dysbiosis

**Fig. 4:**
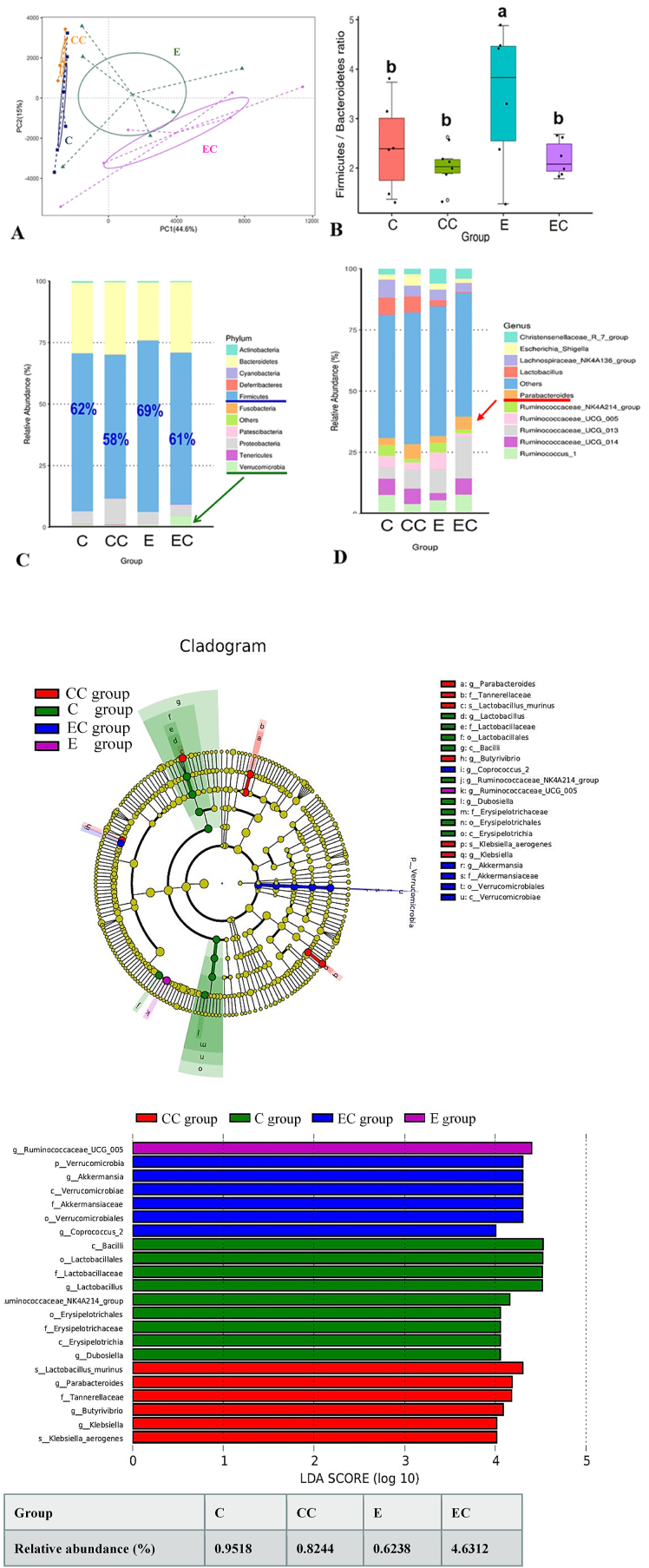
Effect of *C. lentillifera* on the gut microbiota. (A) PCA analysis; (B) Box plots presenting the Firmicutes/Bacteroidetes ratio comparison between groups; top 10 relative abundances of microbial species at the (C) phylum level and (D) generic level in feces of rats; the LEfSe method was used to identify the most differentially enriched taxa in each group. (E) Cladogram and (F) LDA score of bacterial communities among the control (C) group (green), control + CL (CC) group (red), ethanol (E) group (purple), and ethanol + CL (EC) group (blue). Significant difference (p < 0.05) is identified by different letters. (For interpretation of the references to color in this figure legend, the reader is referred to the Web version of this article.)

Development of ethanol-induced liver injury may also result from gut microbiotic dysbiosis (Verdam et al., 2013). The composition of the gut microbiota was determined by next-generation 16S rDNA gene sequencing (NGS). A rarefaction curve is presented in Supplementary Fig. 2, and we used the chao1 and ACE indices to determine community richness in each group. We found that both indices were higher in the EC group compared to the E group, while there were no significant differences between the E and C groups. The PCA also showed that both the ethanol and CL interventions revealed differences in terms of species in the fecal samples (Fig. 4A), indicating that the microbiotic composition differed among the four groups. The *Firmicutes phylum* increased after ethanol administration (E group) compared to the C group, whereas *Firmicutes* relative abundances had returned to a normal ratio in the EC group. Simultaneously, the ratio of *Firmicutes* to *Bacteroidetes* exhibited the same trend. Surprisingly, although the *Verrucomicrobia phylum* showed no significant differences among the C, CC, and E groups, *Verrucomicrobia* relative abundances were significantly elevated in the EC group compared to the E group (Fig. 4B and C). At the genus level, we found that the *Parabacteroides* relative abundance showed a significant increase in the EC group compared to the E group (Fig. 4D). Furthermore, we also utilized an LDA effect size (LEfSe) method to identify the most differentially enriched taxa in each group. Our results showed that p_*Verrucomicrobia* and g_*Akkermansia* of the EC group had significantly increased compared to the E group, while both the C and E groups exhibited no differences in *Akkermansia* relative abundances.

### Ethanol-induced gut barrier dysfunction

**Fig. 5:**
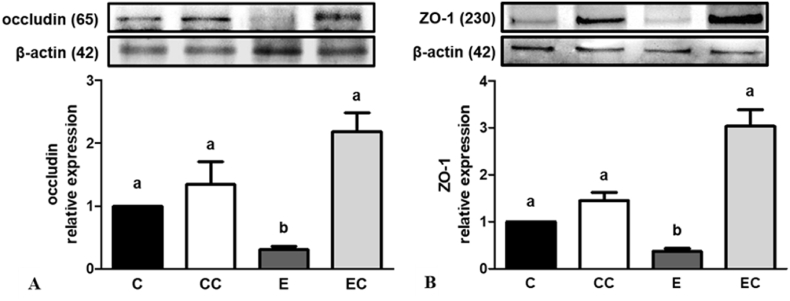
Effect of *C. lentillifera* on intestinal (A) occludin, (B) ZO-1 relative protein expression. Values represent means ± SEM based on 8 rats in each group. Significant difference (p < 0.05) is identified by different letters. C, control group; CC, control + CL group; E, ethanol group; EC, ethanol + CL group.

Excess alcohol intake can also affect intestinal tight junction protein expressions (Turner, 2009). We found that occludin and ZO-1 protein expressions were downregulated under ethanol administration, and both protein expressions had recovered to normal after CL supplementation (Fig. 5). In addition, we evaluated the correlation between the relative abundances of bacterial communities and changes in protein expressions (Supplementary Fig. 3). We found that intestinal occludin and ZO-1 protein expressions were positively correlated with *Akkermansia* (genus) levels. On the other hand, the *Parabacteroides* (genus) relative abundance was negatively correlated with circulatory endotoxins. Interestingly, occludin and ZO-1 protein expressions were also negatively correlated with circulatory endotoxins.

## Discussion

Excess ethanol intake contributes to alcoholic liver injury, and chronic ethanol exposure leads to abnormal lipid metabolism, oxidative stress, and proinflammatory responses that aggravate the progression of ALD (Sugimoto and Takei, 2017). In the present study, we used the modified Lieber & Decarli model to induce ALD (Gustot et al., 2006), and the actual ethanol intake was 9.5 g/kg BW/day (91 g/day for a 60-kg adult in line with the calculation method based on Nair and Jacob, 2016). Abuse drinking can contribute to liver injury and notable elevation of serum AST and ALT. In addition, GGT is also a marker of excessive alcohol consumption which is considered more sensitive in ALD (Peterson, 2004). Our study showed that an ethanol-supplemented liquid diet led to higher AST, ALT and GGT activities, and the pathophysiology report also indicated the presence of increased steatosis and liver inflammation (Fig. 1 and Table 1). Conversely, CL treatment diminished increases in the ethanol-induced liver index and hepatic inflammation score. These results indicate the successful establishment of our ALD model and the potential of CL to protect against ethanol-induced liver injury.

Oxidative stress is a crucial factor in promoting ALD progression (Shi et al., 2017). CYP2E1 is an enzyme responsible for ethanol metabolism during chronic alcohol exposure which also increases the formation of ROS. Additionally, the lipid peroxidation byproduct, MDA, also increases due to the microsomal ethanol-oxidizing system (Galicia-Moreno et al., 2016). Recent evidence demonstrated that polysaccharides from marine algae contribute to their antioxidative properties of reducing oxidative stress (Sun et al., 2014). *C. lentillifera* was also reported to show strong hydrogen peroxide scavenging activity due to its phenolic compounds *ex vivo* (Nguyen et al., 2011), and immunomodulatory activities through increasing phagocytosis of macrophage cells as a result of its polysaccharide content (Maeda et al., 2012). Although ethanol intake significantly increased hepatic MDA levels and CYP2E1 protein expression, we found a slightly, but not significantly, lower trend for both hepatic MDA and CYP2E1 after 12 weeks of CL supplementation. Thus, these results suggested that protective effects of CL in the present study cannot thoroughly be explained by its antioxidative activities.

Evidence has demonstrated that ethanol consumption leads to enteric dysbiosis and gut barrier dysfunction, which allows endotoxins into the portal circulation owing to the increased intestinal permeability and even gut leakiness (Altamirano and Bataller, 2011), and this consequently increases the production of proinflammatory cytokines such as TNF-α and IL-1β by activating Kupffer's cells (Gustot et al., 2006). Consistent with a previous study, we found that the inflammation score, endotoxin levels, and proinflammatory cytokines were elevated after 12 weeks of ethanol administration, and CL supplementation improved the above effects. In the liver, endotoxin interacts with the TLR4 receptor and triggers the MyD88-dependent or -independent (TRIF) pathway, and activates NF-κB p65 dissociation from IκBα and its translocation into nuclei in order to upregulate the transcription of related inflammatory genes, which accelerates ALD progression (Ceni et al., 2014; Neuman et al., 2015). In the present study, our data demonstrated that CL supplementation blunted ethanol-induced TLR4 pathway protein expression and the increment in the p–NF–κB-to–NF–κB ratio. These results suggested that CL may improve ethanol-induced hepatic inflammation via modulating the lipopolysaccharide (LPS)-TLR4 signaling pathway. In addition, some studies demonstrated that there might be sex dependent disparity in inflammatory liver diseases (Guy and Peters, 2013). Thus, the possible impact of gender differences may need further study to clarify.

Bidirectional communication between the gut and liver, also called the gut-liver axis, linked through the portal vein and bile duct has recently garnered attention by virtue of being highly related to ALD progression (Tripathi et al., 2018). Therefore, we analyzed the microbiotic composition in each group, and results exhibited higher taxa richness after CL supplementation. Furthermore, both ethanol administration and CL treatment resulted in differences in species in fecal samples. Then, we evaluated the relative abundances of bacterial communities in rats under chronic ethanol consumption and observed significantly higher *Firmicutes phylum* abundance in the E group which is inconsistent with a previous study reporting that ethanol reduced abundance of the genus *Lactobacillus* of the *Firmicutes* (Hartmann et al., 2012). Nonetheless, after further analysis, we observed that the significantly higher abundance of the *Firmicutes phylum* in the E group was due to elevation of the family *Clostridiaceae*. In alcoholic patients with pancreatitis, researchers also found higher levels of the family *Clostridiaceae* (Ciocan et al., 2018), which may be related to poor metabolic outcomes (Bermingham et al., 2017). Additionally, we also found that ethanol consumption increased the F-to-B ratio, whereas it changed to a normal ratio after CL supplementation. The F-to-B ratio is regarded as dysbiosis, whereby the former is usually observed with obesity, and the latter with inflammatory bowel disease (Stojanov et al., 2020). The F-to-B ratio was observed to be higher in vivo on a high-fat diet model and was correlated to metabolic disorders (Verdam et al., 2013; Zhu et al., 2013).

*Parabacteroides goldsteinii* was proven to be associated with changes in the intestinal permeability. Studies showed that *Akkermansia muciniphila* could bind to gut epithelial cells which improved a leaky gut (Anhê and Marette, 2017). Treatment with *A. muciniphila* for 5 weeks not only reversed high fat diet-induced obesity, but also decreased circulating endotoxin levels (Plovier et al., 2017). The intestinal epithelial barrier is composed of epithelial cells, tight junction proteins such as occludin and ZO-1, and various biochemical elements (Turner, 2009). It has been shown that excess ethanol consumption impairs tight junction protein expression (Wang et al., 2014). Interestingly, we observed that the genera *Parabacteroides* and *Akkermansia* were elevated after CL supplementation during the 12 weeks of ethanol administration. Thus, we further analyzed intestinal barrier protein expressions and found that occludin and ZO-1 protein expressions were downregulated in the ethanol-fed group. In contrast, CL supplementation reversed the decrement in tight junction protein expressions induced by the ethanol liquid diet, and no difference was found among the C, CC, and EC groups. We also found that the g_*Parabacteroides* abundance was negatively correlated with circulatory endotoxin levels, and g_*Akkermansia* abundance was positively correlated with intestinal occludin and ZO-1 protein expressions (Fig. 5). These results suggested that CL may exhibit a prebiotic-like effect to improve dysbiosis and strengthen the intestinal barrier function.

Abnormal very-low-density lipoprotein (VLDL) synthesis, β-oxidation decrement, and endogenous lipid accumulation in the liver occurred under continual alcohol consumption which brought about steatosis (Lieber, 2004). Moreover, chronic alcohol abuse impairs hepatic lipid hemostasis-related protein expressions, such as SREBP1 and PPAR-α (Sozio and Crabb, 2008). SREBP1 is required for de novo lipogenesis in the liver, and regulates downstream acetyl-CoA carboxylase (ACC) and fatty acid synthase (FAS) gene transcription (You et al., 2002). On the other hand, PPAR-α is a transcription factor that binds with the retinol X receptor (RXR) to regulate lipid metabolism in the liver (Galli et al., 2001). Algae contain abundant polysaccharides, generally considered beneficial for modulating lipid metabolism (Mohamed et al., 2012). A previous study showed that CL exhibits potent antihyperlipidemic properties by decreasing serum TC and TGs in a high-fat diet model (Gara et al., 2017). However, despite increases in hepatic TC and TG concentrations and SREBP1 expression in the ethanol-fed group, we found no differences in hepatic lipid levels, steatosis scores, or SREBP1 expressions between the E and EC groups. Therefore, the hepatoprotective effects of CL in this study cannot be completely explained by regulating lipid metabolism, and different experimental designs and models may be one of the reasons for the diverse results.

## 5 Conclusions

In conclusion, our results indicated that dietary CL supplementation improved ethanol-induced liver injury, and three of the possible mechanisms may be through strengthening the intestinal barrier function, alleviating dysbiosis, and modulating the TLR4 pathway, which may alleviate ALD progression.

## Author Contributions

Kuan-Yu Lin: Conceptualization, Investigation, Writing – original draft preparation, Project administration. Hsin-Yi Yang: Conceptualization. Suh-Ching Yang: Conceptualization, writing and editing, Project administration. Ya-Ling Chen: Investigation. Y Watanabe: Project administration. Jiun-Rong Chen: writing and editing, Funding acquisition. All authors have seen and approved the contents of the submitted manuscript.

## Declaration of competing interest

This research received no specific grant from any funding agency and the authors declare no conflicts of interest.

## Data Availability

No data was used for the research described in the article.
