# Supplementary material for: Caulerpa lentillifera improves ethanol-induced liver injury and modulates the gut microbiota in rats
Source: Curr Res Food Sci. 2023 Jul 8;7:100546. doi: 10.1016/j.crfs.2023.100546 (PMC10362798; doi:10.1016/j.crfs.2023.100546)
Supplement: Multimedia component 1 [file mmc1.pdf]

## *Supplementary results*

**Supplementary Table 1**

Experimental liquid diet formula of different groups.

| Ingredients (g/L) | C | CC | E | EC |
|---|---|---|---|---|
| Casein | 41.4 | 41.4 | 41.4 | 41.4 |
| L-cystine | 0.5 | 0.5 | 0.5 | 0.5 |
| Soy oil | 48.5 | 48.5 | 48.5 | 48.5 |
| Dextrin-maltose | 98.0 | 98.0 | 13.5 | 13.5 |
| Choline bitartrate | 0.5 | 0.5 | 0.5 | 0.5 |
| Cellulose | 10.0 | 10.0 | 10.0 | 10.0 |
| Xanthan gum | 3.0 | 3.0 | 3.0 | 3.0 |
| Vitamin mixture | 2.2 | 2.2 | 2.2 | 2.2 |
| Mineral mixture | 7.7 | 7.7 | 7.7 | 7.7 |
| Ethanol | - | - | 48.3 | 48.3 |
| *C. lentillifera* (CL) | - | 8.4 | - | 8.4 |

The experimental liquid diet was prepared based on modified Lieber-Decarli ethanol liquid diet and the control liquid diet replace ethanol with isocaloric dextrin-maltose (Lieber & Decarli, 1994). Casein high nitrogen, L-cysteine, choline bitartrate, cellulose, mineral mixture (AIN-93M), vitamin mixture (AIN-93M) were acquired from ICN biochemical (Aurora, OH, USA) Soybean oil was obtained from Taiwan Sugar Corporation (Tainan, Taiwan). Dextrin-maltose was obtained from Ingredion Incorporated (Westchester, IL, USA). Xanthan gum was purchased from Sigma (SI. Louis, MO, USA). Dried *C. lentillifera* powder was kindly provided by East Green (Hualian, Taiwan). C, control group; CC, control + CL group; E, ethanol group; EC, ethanol + CL group.

**Supplementary Table 2.**

Body weight and average daily food in rats under chronic ethanol consumption

| | C | CC | E | EC |
|---|---|---|---|---|
| Body weight (g/d) | | | | |
| Initial | 216.8 ± 2.5 | 218.6 ± 1.9 | 218.6 ± 1.7 | 218.6 ± 2.3 |
| Final | 502.2 ± 8.5 $^{a}$ | 534.5 ± 10.8 $^{a}$ | 447.5 ± 8.6 $^{b}$ | 460.5 ± 8.0 $^{b}$ |
| Food intake (kJ/d) | 319.6 ± 0.0 | 319.6 ± 0.0 | 320.5 ± 1.9 | 320.8 ± 0.9 |
| Ethanol intake (g/d) | - | - | 3.7 ± 0.5 | 3.8 ± 0.2 |
| CL intake (g/d) | - | 0.7 ± 0.0 | - | 0.7 ± 0.0 |

Values represent means ± SEM and n = 8. Significant difference ($p < 0.05$) is identified by different letters. C, control group; CC, control + CL group; E, ethanol group; EC, ethanol + CL group; CL, *C. lentillifera*.

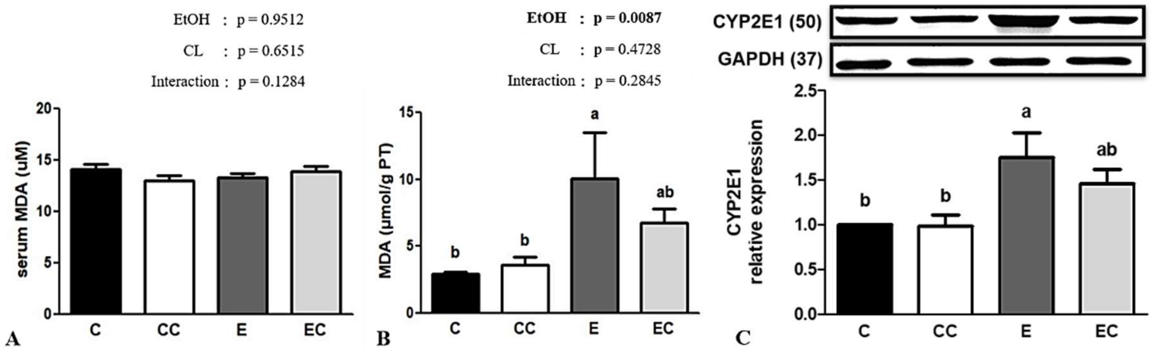


**Supplementary figure 1. Effect of Caulerpa lentillifera (CL) on (A) serum malondialdehyde (MDA) levels, (B) hepatic MDA level and (C) cytochrome P450 2E1 (CYP2E1) relative protein expression analyzed by western blotting.** Values represent means ± SEM and n = 8. Significant difference ($p < 0.05$) is identified by different letters. C, control group; CC, control + CL group; E, ethanol group; EC, ethanol + CL group; CYP2E1, cytochrome P450 2E1; MDA, malondialdehyde.

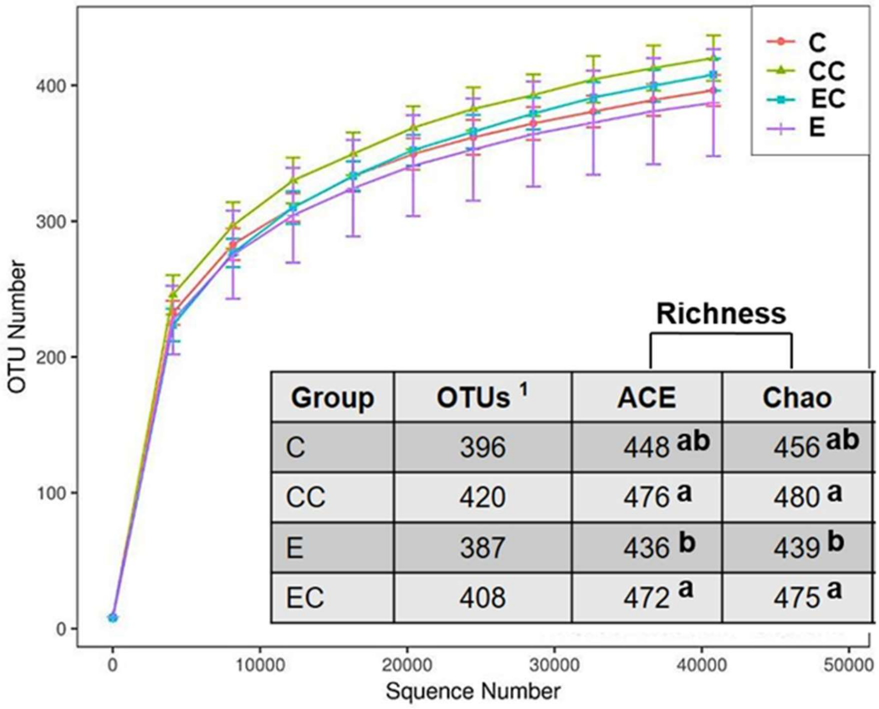


| Group | OTUs [1] | ACE | Chao |
|---|---|---|---|
| C | 396 | 448 ab | 456 ab |
| CC | 420 | 476 a | 480 a |
| E | 387 | 436 b | 439 b |
| EC | 408 | 472 a | 475 a |

[1]OTU picking: 97% sequence similarity
OTU, operational taxonomic unit;
ACE, Abundance-based Coverage Estimator

**Supplementary figure 2. PCA analysis showing differences in terms of species in fecal samples.**

Alpha diversity was on rarefaction curve. Significant difference ($p < 0.05$) is identified by different letters. C, control group; CC, control + CL group; E, ethanol group; EC, ethanol + CL group.

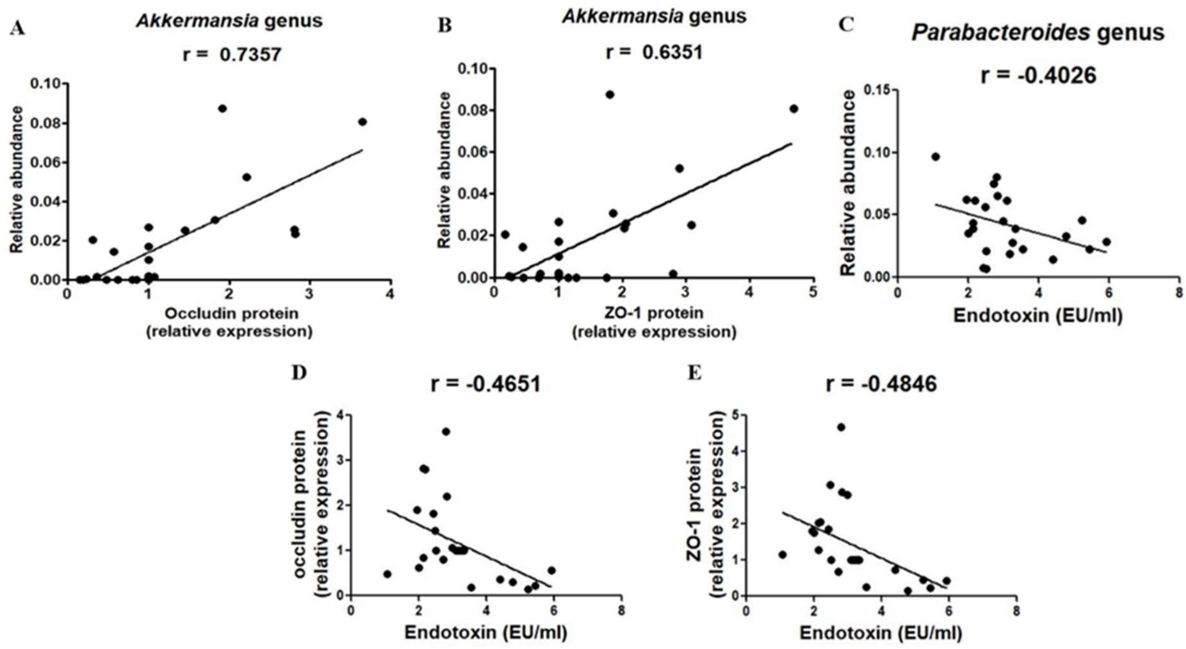


**Supplementary Figure 3. Linear regression plot**

(A) linear regression of occludin relative protein expression correlated with Akkermansia, (B) ZO-1 relative protein expression correlated with Akkermansia, (C) circulation endotoxin levels correlated with Parabacteroides, (D) circulation endotoxin levels correlated with occludin relative protein expression, (E) circulation endotoxin levels correlated with ZO-1 relative protein expression. $P < 0.05$ and Pearson's correlation coefficient (r) > 0.4.
